# 

**Published:** 1913-12-15

**Authors:** 


### ANNOUNCEMENTS.

### THE PANAMA PACIFIC DENTAL CONGRESS

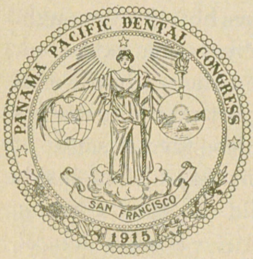

OPENS AUG. 30TH, 1915

The work of organizing the Panama-Pacific Dental Congress is progressing in a most satisfactory manner, and it is confidently expected that by January 1, 1914, twenty months before the opening of the Congress, all the preliminary work of organization will be accomplished.

A few foreign countries and a few of our States have yet to appoint Executive Committees to carry on their part of the work of publicity and securing program and membership. Within the next two weeks invitations will be sent to those who are selected by the Committee of Organization to act as officers of the various sections of the Congress, and in each case an urgent request will be made for a prompt reply, that there may not be experienced in this matter the delay which in some cases has attended the appointment of State and National Executive Committees.

Three hundred thousand "stickers" bearing the seal of the Congress and the date on which it will convene have been sent to dealers in dental and pharmaceutical preparations throughout the world, all of whom have expressed a willingness to attach them to every package and letter sent to their customers between now and August 30th, 1915. Demands are already being made for more "stickers" and probably one hundred thousand more will be distributed. The Congress will in this way be brought to the attention of every dentist in the world, not once, but many times, and no one will be allowed to forget the date on which he should be in San Francisco to participate in what promises to be the world's greatest Dental Congress.

Work is progressing rapidly on the Auditorium in which the Congress will meet, and it will undoubtedly be housed in one of the largest and most complete buildings ever erected for such a purpose.

The Congress will be in keeping with the Exposition of which it forms a part, and every effort will be made to provide for the comfort and entertainment of every member. That work on the buildings for the Exposition is advancing rapidly may be noted from the fact that Machinery Hall is now over 85 per cent. finished. This building is 968 feet long and 368 feet wide. Over 7,500,000 feet of lumber has been used in its construction, and it is the largest frame building in the world.

### SIXTH INTERNATIONAL DENTAL CONGRESS.

London, August 3d to 8th, 1914.

SECTIONS OF MUSEUM:

1. Dental Anatomy, Histology and Physiology. 2. Dental Pathology and Bacteriology. 3. Dental Surgery and Therapeutics. 4. Dental Physics, Chemistry, Radiography and Metallurgy. 5. Dental Prosthesis. 6. Orthodontics. 7. Oral Surgery and Surgical Prosthesis. 8. Anesthesia. 9. Oral Hygiene, Public Instruction and Public Dental Services. 10. Dental Education.

It is intended that the Museum or Exhibit shall be an International Collection of objects of interest, and be representative of every Section of the Congress. Its Nature and Scope include the following:—

1. Specimens showing the Evolution of Tooth Forms and of the Dentition of Man. Histological Preparations bearing upon recent research. Exhibits illustrating the Chemical Composition and Physiological Action of the Saliva.

2. Specimens of Morbid Conditions of the Teeth, Palate, Gums and Jaws, such as Odontomes, Dental and Dentigerous Cysts, New Growths, Diseases of the Periodontal Membrane, etc., Photomicrographs of Oral Micro-Organisms, and

Cultures of Micro-Organisms, in Test Tubes or in Petri Dishes. New Bacteriological Apparatus and Appliances.

3. Specimens of Teeth, Gums and Jaws affected by "Pyorrhea Alveolaris." Microscopical and Lantern Slides of the same. Exhibits of Various New Methods of "Inlaying" Cavities in Teeth. Exhibits of New Methods of "Crowning" Teeth.

4. Radiographs of the Normal Dental Tissues, and of Diseases of the same and Associated Parts.

5. Exhibition of various kinds of Articulators. Specimens showing the various Methods of "Pressure Casting." Specimens showing modern forms of Continuous-Gum Work.

6. Models showing Abnormalities in position of the Teeth, and Appliances for the correction of the same.

7. Specimens illustrating Methods of Dealing with Surgical Conditions of the Teeth and Jaws, including Cleft Palate, Harelip, Fracture and Resection of the Jaws.

8. Specimens illustrating the History and Evolution of Anesthesia.

9. Photographs, Charts, Diagrams, Specimens and Statistics of School Clinics. Methods of the Instruction of the Public in the principles of Oral Hygiene.

10. Instruction Forms, Charts, Diagrams, Specimens and Demonstration Models used in relation to Dental Education. The Specimens will include those employed for teaching purposes, and also Specimens of Work of both Students and Pupils, completed in accordance with the definite courses given.

11. Historical Objects of Interest, such as Books, Instruments, Pictures, etc.

REGULATIONS.

1. Intending exhibitors must give full information with regard to the exhibits suggested for inclusion in the Museum on the application form supplied overleaf. Exhibitors are requested to *send in the Application Forms as early as possible.* Those received after June 30, 1914, cannot be considered, and lists of exhibits sent after this date cannot be printed in the Official Catalogue.

2. All exhibits must be received, properly packed (see below), registered and carriage paid, at the University of London, South Kensington, London, S. W., on or after Monday, July 13th, and before July 20th, 1914.

3. The Committee has the absolute right of acceptance or refusal of the exhibits.

4. The Committee reserves to itself the right of arranging the exhibits.

5. The Committee will be responsible for the proper exhibition of the several exhibits, and for their secure packing and dispatching on return. All exhibits will be insured. and any claim for compensation for damage or loss must be made within three months of the closing of the Museum.

6. Any exhibitor making a communication to a Section may arrange with the Hon. Curator of that Section for his specimens to be available at the Sectional Meetings, in which case the exhibitor shall be responsible for the safe return of his exhibit uninjured.

7. No specimen may be removed from the Museum cases without the Consent of the Committee.

INSTRUCTIONS FOR SENDING SPECIMENS, ETC.

A. All specimens of a brittle nature should be *securely packed* in wooden boxes, *on the lid of which the name and address of the exhibitor should be painted or written.*

On the inside of the lid a written list of the contents should be affixed.

B. In the case of wall diagrams and other objects of a non-brittle nature, parcels should be made, and the inscriptions should be affixed to the paper covering as under A.

C. *Each specimen should have some distinctive label attached to it, in order that it may be identified, and should be numbered according to the Section of the Museum in which it is to be placed.*

D. The box should have a large label nailed on, with the address as follows, legibly written upon it:—

For full particulars address,

Mr. F. N. Doubleday, *Hon. Secretary,*

International Dental Museum, University of London,
South Kensington, London, S. W.

M^cSwain

### DENTAL RELIEF FUND.

OFFICE OF DR. EDWARD S. GAYLORD,
63 Trumbull St., New Haven Conn.

The Committee on the National Dental Association Relief Fund, are, this year, instituting a more active campaign than has been followed since its appointment two years ago at the annual meeting in Cleveland, to solicit financial support, to the extent of establishing a National Dental Relief Fund, the interest of which may be dispensed to members of the N. D. A. in good standing, who by permanent disability, are unable to support themselves in the practice of their profession, and those dependent upon them.

We are to issue immediately, one million Christmas Seals of suitable design, to be used on all forms of correspondence or otherwise, and beg the assistance of the different dental depots or supply houses, to aid the Relief Fund Committee (without compensation) in facilitating the sale of these seals which is in every sense a work of charity.

Strict account of all disbursements and sales will be kept, and none but actual expense in printing and postage will be charged against the Fund.

All receipts from the sale of Seals, the price of which has been set at $1.00 per hundred, should be forwarded to this office, when they will be turned over to Dr. H. B. McFadden, Treasurer of the National Dental Association, requiring his receipt for same. With the above described charitable object in view, are you willing to aid us? Posters of suitable design, announcing the sale will be supplied with shipment of the Seals (to wit):

Christmas Seals
For the Benefit of the
National Dental Association Relief Fund
For Sale Here.

If willing to co-operate with us, how many seals may I send you? Very respectfully,

L. G. Noel, Wm. T. Chambers,
James McManus, E. S. Gaylord,
*National Relief Fund Committee.*

